# Anabolic and catabolic responses to different modes of exercise in patients with chronic kidney disease

**DOI:** 10.1186/s12882-026-04891-4

**Published:** 2026-03-13

**Authors:** Douglas W. Gould, Luke A. Baker, Thomas J. Wilkinson, Nicholas Eastley, Robert U. Ashford, Matthew Denniff, Matthew Graham-Brown, João L. Viana, Andrew Philp, Emma L. Watson

**Affiliations:** 1 Intensive Care National Audit and Research Centre, London, UK; 2 Division of Respiratory Sciences, College of Life Sciences, University of Leicester, Leicester, UK; 3 Leicester Biomedical Research Centre, Leicester Diabetes Centre, University of Leicester, Leicester, UK; 4 Leicester Orthopaedics, University Hospitals of Leicester, Leicester, UK; 5 Division of Cancer Sciences, College of Life Sciences, University of Leicester, Leicester, UK; 6 Division of Cardiovascular Sciences, College of Life Sciences, University of Leicester, Leicester, UK; 7 Leicester Kidney Lifestyle Team, Division of Cardiovascular Sciences, University of Leicester, Leicester, UK; 8 Research Center in Sports Sciences, Health Sciences and Human Development, CIDESD, University of Maia, Porto, Portugal; 9 Centre for Healthy Ageing, Centenary Institute, Sydney, New South Wales Australia; 10 School of Sport, Exercise and Rehabilitation Sciences, University of Technology Sydney, Sydney, New South Wales Australia; 11 Department of Cardiovascular Sciences, Centre of Sarcopenia and Muscle Wasting, Clinical Sciences Wing, Glenfield Hospital, University of Leicester, Leicester, LE3 9QP UK

**Keywords:** Chronic kidney disease, Exercise, Biopsy, Anabolic, Catabolic

## Abstract

**Background:**

Muscle wasting is common in chronic kidney disease (CKD), contributing to impaired function and poor health outcomes. Exercise is recommended, but the molecular responses of skeletal muscle to different exercise modalities remain unclear. This study examined anabolic, catabolic, and myogenic responses to aerobic exercise (AE) versus combined exercise (CE; aerobic plus resistance) in CKD.

**Methods:**

Muscle biopsies were collected from participants in a 12-week randomized controlled trial (the ExTra CKD trial) at baseline, 24 hours after initial exercise bout (untrained), and 24 hours after the final session (trained). Western blotting and RT-qPCR assessed markers of protein synthesis, degradation, and regeneration. Complementary in vitro experiments used mechanically stretched primary skeletal muscle cells from CKD patients and healthy controls to investigate the temporal dynamics of anabolic signalling.

**Results:**

AE did not alter Akt phosphorylation at any time point (*p* > 0.05). In contrast, CE showed no acute effect before training (*p* > 0.05) but significantly increased Akt phosphorylation after training (+118% vs baseline, *p* = 0.02), indicating partial restoration of anabolic signalling. CE also attenuated the acute increase in the 14-kDa actin fragment observed in the untrained state (+253%, *p* = 0.04), and increased expression of myogenic marker MyoD following training (*p* = 0.01), whereas AE effects were minimal (*p* > 0.05). In vitro mechanical stretch induced marked increases in Akt (CKD: +5651%, *p* = 0.012; healthy control: +3437%, *p* = 0.028) and p70S6K phosphorylation immediately post-stretch (CKD: +1076%, *p* = 0.014; healthy control: +712%, *p* = 0.038). No significant differences in the temporal signalling response were observed between CKD and healthy control cells (P-Akt: *p* = 0.84; P-P70S6K: *p* = 0.052).

**Conclusion:**

CE, but not AE alone, elicits beneficial anabolic and myogenic adaptations in skeletal in CKD. Combining in vivo and in vitro approaches offers deeper mechanistic insight into exercise-induced molecular adaptations and highlights the importance of including resistance training to overcome anabolic resistance in this population.

**Trial registration:**

This study includes analysis of tissue samples from two clinical trials both registered with the ISRCTN- ExTRa CKD: ISRCTN (no. 36489137, registered 21/04/2014) and Explore CKD: ISRCTN (No. 18221837, registered 10/02/2016).

**Supplementary information:**

The online version contains supplementary material available at 10.1186/s12882-026-04891-4.

## Introduction

Chronic kidney disease (CKD) is a global public health emergency with an estimated prevalence of 9.1% [1]. A common complication at all stages of CKD is muscle wasting, which contributes to reduced physical function independent of age [2]. This loss of muscle is associated with poor clinical outcomes; for example, diagnosed sarcopenia has been shown to increase the risk of all-cause mortality threefold [3] (authors defined sarcopenia as reduced handgrip strength (<30th percentile for age and sex) in combination with low muscle mass assessed by either Mid-Arm Muscle Circumference (MAMC) <90% of reference, muscle wasting on Subjective Global Assessment (SGA), or reduced skeletal muscle mass index by Bioelectrical Impedance Analysis (BIA) (<10.76 kg/m^2^ in men; <6.76 kg/m^2^ in women)). Furthermore, individuals with CKD exhibit high levels of physical inactivity [4], which is also an independent risk factor for mortality [5].

Although regular physical activity is strongly recommended for individuals with CKD, and formal exercise guidelines have recently been published for this population [6], structured exercise programmes remain underutilised and rarely form part of clinical care. For example, less than one fourth of dialysis units provide exercise programmes for dialysis patients [7]. Importantly, physical inactivity itself contributes to muscle wasting, and likely exacerbates CKD-related muscle loss. To maximise the benefits of exercise training, it is crucial to understand the underlying molecular mechanisms, particularly how different types of exercise affect skeletal muscle homeostasis. This knowledge could guide the development of adjunctive strategies, such as targeted nutritional or pharmacological interventions. While a few studies have begun to explore this area in humans [8–10], a comprehensive understanding of these mechanisms in the CKD population remains limited.

Although the mechanisms that drive muscle wasting in CKD have not been fully elucidated, the fundamental physiological processes governing skeletal muscle size regulation are well characterized, and are likely to play a role in the development of muscle wasting here. Skeletal muscle mass is maintained by a dynamic balance between protein synthesis and degradation. Anabolic signalling through the Akt/mTOR pathway plays a central role in promoting muscle protein synthesis, while catabolic pathways, including the ubiquitin–proteasome system and myostatin signalling, regulate protein breakdown. In CKD, this balance is often disturbed, exacerbated by systemic factors such as insulin resistance, inflammation, and metabolic acidosis [11–13]. Impairments in anabolic signalling, particularly the reduced phosphorylation of Akt and downstream targets, have been observed in animal models of CKD and are associated with muscle atrophy and impaired regeneration. While resistance exercise is known to activate these anabolic pathways in healthy individuals, the extent to which this occurs in those with CKD remains under investigation.

Our previous work has shown that in CKD, the expected anabolic responses to resistance exercise may be blunted, consistent with the concept of “anabolic resistance” [10]. we observed attenuated activation of the insulin signalling pathway and limited upregulation of myogenic markers in patients with CKD after a single bout of resistance exercise. However, some of these blunted responses appeared to normalise following a period of training, a finding that has yet to be replicated. In addition, we have also reported attenuated mitochondrial adaptations to both aerobic and combined exercise in this population [8], an effect that was not improved with exercise training. This raises the possibility that aerobic exercise alone may be insufficient to elicit optimal skeletal muscle remodelling in CKD.

Building on this previous work, the aim of this study was to investigate the post-exercise anabolic and catabolic responses of skeletal muscle to aerobic versus combined exercise in individuals with CKD, comparing these responses before and after a structured training period. We hypothesised that the inclusion of resistance training to aerobic training would promote a more favourable balance between anabolic and catabolic signalling pathways compared with aerobic exercise alone, both before and after a period of training. To achieve this, we used muscle biopsies collected from the previously published ExTra CKD randomised controlled trial [14]. In addition to analysing these in vivo responses, we used an in vitro mechanical stretch model to better understand the temporal dynamics of mechanically induced molecular signalling in primary skeletal muscle cells from CKD and healthy donors. Together, these complementary approaches provide a comprehensive view of how skeletal muscle in CKD responds to exercise and highlight important considerations for optimising interventions aimed at improving muscle health in this vulnerable population.

## Materials and methods

This study is a secondary analysis of the previously reported ExTra CKD randomised controlled trial [14] and includes complementary in vitro investigations using primary cells obtained from participants in the Explore CKD study.

### The ExTra CKD trial

In brief, 54 non-dialysis patients with CKD stages 3b–5 were enrolled in a 12-week, thrice-weekly supervised exercise intervention and randomised to either combined exercise (CE) or aerobic exercise (AE) alone. Participant characteristics are shown in Table 1. Each participant served as their own control during a six-week run-in period prior to randomisation. As reported previously, no changes were reported in any physiological outcome during this period [13]. The present study is a secondary mechanistic analysis of skeletal muscle biopsies desgined to compare molcular responses between exercise modalities rather than exercise vs no exercise. The AE intervention consisted of circuit-based aerobic activities such as treadmill walking or running, cycling, and rowing. The CE group undertook the same aerobic training, with the addition of resistance exercises performed during two of the three weekly sessions. Participants aimed to complete 30 minutes of moderate-intensity aerobic exercise at 70–80% of their maximum heart rate, which was determined by a maximal exercise tolerance test. For the resistance component, participants performed 3 sets of 12–15 repetitions of leg extensions at 70% of their estimated one-repetition maximum, which was determined from a five-repetition maximum test. The order of aerobic and resistance exercise within each combined training session was not rigidly standardised. In most sessions, participants completed the aerobic component first, followed by the resistance exercise component, reflecting pragmatic delivery of the intervention. When patients could comfortably perform 3 sets with good form the training load was increased. Exclusion criteria for the ExTra CKD trial included: age < 18 years, physical impairment sufficient to prevent undertaking the intervention, recent myocardial infarction, unstable chronic conditions, or an inability to give informed consent, and a BMI > 40 (due to difficulties in muscle size measurement). Diabetic patients were included if haemoglobin A1C was < 9%. Biopsy specific exclusion criteria included: blood thinning medication and platelet disorders.

**Table 1:** Participant characteristics

	Aerobic Exercise (*n* = 10)	Combined Exercise (*n* = 9)
Age (years)	65 ± 8	59 ± 18
Gender (*n* men/women)	4/6	3/6
Ethnicity	White British *n* = 9 Black Caribbean *n* = 1	White British *n* = 8 South Asian *n* = 1
eGFR (ml/min/1.73 m^2^)	27 ± 9	27 ± 6
Serum Bicarbonate (mmol/l)	25 ± 6	26 ± 5
BMI (kg/m^2^)	30 ± 6	30 ± 5
Creatinine (μmol/L)	209 ± 75	218 ± 69
Urea (mmol/L)	12 ± 4	13 ± 5

Abbreviations: BMI, body mass index; eGFR, estimated glomerular filtration rate. Data are mean ± SD

**Fig. 1:**
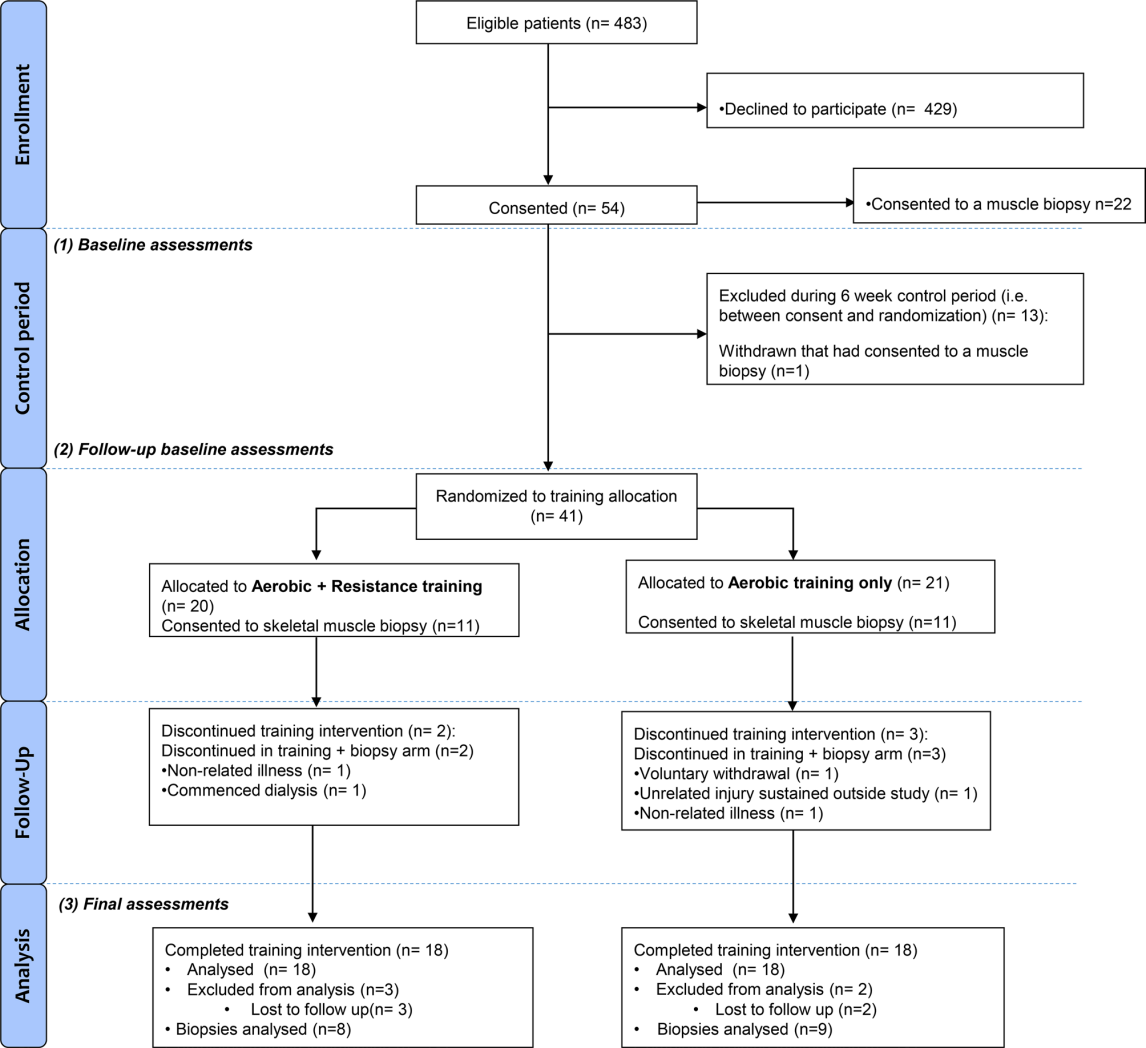
CONSORT flow diagram for the exercise training trial and muscle biopsy sub-study. Consolidated Standards of Reporting Trials (CONSORT) flow diagram illustrating participant recruitment, allocation, follow-up, and analysis for the parent exercise training trial, with extension to the muscle biopsy sub-study reported in this manuscript. The diagram shows the number of participants screened, consented, randomised to aerobic exercise or combined aerobic and resistance exercise training, and completing the intervention, as well as reasons for withdrawal or exclusion at each stage

A subset of participants (AE, *n* = 10; CE, *n* = 9) consented to skeletal muscle biopsies of the vastus lateralis which were collected using an 11 g ACECUT automatic biopsy needle (TSK Laboratory, Netherlands) as previously described [10]. Samples were collected in a fasted state at three time points: baseline, 24 hours after the first training session (untrained), and 24 hours after the final session (trained). Following the removal of visible adipose tissue, biopsies were snap-frozen in liquid nitrogen and stored for later analysis. Participation in the biopsy component was optional, and all participants in the parent trial were approached and invited to consent. Consequently, the final sample size reflects a pragmatic number determined by participant willingness, ethical considerations, and the feasibility of repeated muscle biopsy sampling in a clinical population, rather than a statistically defined target. The flow of participants through the study is shown in Fig. 1. The study was given favourable ethical opinion by the National Research Ethics Committee, East Midlands, Leicester South (Ref: 13/EM/0344). All patients gave written informed consent and the trial was conducted in accordance with the Declaration of Helsinki. This study is registered with the ISRCTN (Ref: 36489137).

### The explore CKD study

A single lower leg skeletal muscle biopsy was obtained from six individuals with CKD and eight age- and sex-matched non-CKD controls as part of the Explore CKD study (participant characteristics are detailed in Table 2). CKD participants were recruited from outpatient clinics at Leicester General Hospital, UK, between 1^st^ January 2016 and 2^nd^ November 2020. Healthy controls (HC) with no significant medical history were recruited from orthopaedic theatre lists during procedures for benign tumour removal. Biopsies were collected during the scheduled surgical procedure and a small piece of muscle tissue was removed from the same area that the operation was being performed on using a surgical blade. Muscle biopsies from CKD participants were collected using an 11 g ACECUT automatic biopsy needle (TSK Laboratory, Netherlands). All biopsies were collected into 5 mL Hams F10 media (ThermoFisher, UK, 11550043) containing 1% penicillin streptomycin and 1% Gentamycin, and placed on ice for two hours prior to satellite cell isolation. Cells were isolated as described below. The study was given favourable ethical opinion by the National Research Ethics Committee, East Midlands, Leicester South (Ref: 15/EM/0467). All patients gave written informed consent and the trial was conducted in accordance with the Declaration of Helsinki. The Explore CKD study is registered with the ISTCTN (ISRCTN18221837).

**Table 2:** Skeletal muscle biopsy donors for primary cell culture establishment

	Healthy Controls (*n* = 8)	CKD Patients (*n* = 6)
Age (years)	61 ± 14	63 ± 6
Gender (*n* men/women)	4/4	2/3
Ethnicity	White British *n* = 8	White British *n* = 6
eGFR (ml/min/1.73 m^2^)	25.0 ± 4.7	82.0 ± 9.9
BMI (kg/m^2^)	28 ± 6	29 ± 5
Physical activity status (GPPAQ result)	Inactive	Inactive

Abbreviations: eGFR, estimated glomerular filtration rate. Data are mean ± SD

### Molecular biology techniques

#### Satellite cell isolation procedure

Satellite cells were isolated and propagated as previously described [15]. Briefly, fresh muscle tissue was washed three times in HamsF10 (ThermoFisher, UK, 11550043) (containing 1% penicillin streptomycin ThermoFisher, UK, 15140122, and 1% Gentamycin ThermoFisher, UK, 15750060), minced into small fragments, and enzymatically digested in two incubations with collagenase IV (1 mg/mL, ThermoFisher, UK, 10780004), bovine serum albumin (BSA, 5 mg/mL, Merck, UK, 810685) and trypsin (500 µl/mL, ThermoFisher, UK, 11580626) at 37 °C with gentle agitation. The resultant supernatant was strained through a 70 µm nylon filter and centrifuged at 800 g for 7 mins. The cells were washed in Hams F10 with 1% penicillin-streptomycin and pre-plated on uncoated 9cm2 petri dishes in 3 mL growth medium (GM; Hams F10 Glutamax, ThermoFisher, UK, 11550043; 20% Foetal bovine serum, FBS, Merck, UK, F7524; 1% penicillin streptomycin, ThermoFisher, UK, 15140122; 1% fungizone, Merck, UK, A2411) for 3 h. The cell suspension was then moved to collagen I coated 25 cm2 flasks and kept in standard culture environmental conditions (37 °C, 5% CO2). For the expansion of satellite cell populations, cells were grown to approximately 70% confluence in GM that was changed every other day. Cells were subsequently trypsinised and counted using the trypan blue exclusion method (ThermoFisher, UK, 15250061) before being frozen down for later use.

#### Cell culture and in vitro mechanical stretch

Myoblasts were seeded on 6-well Flexcell culture plates (Flexcell International Corp, NC, USA) and cyclic multiaxial stretch applied using a Flexcell F×3000 system set to 2 second sine wave stretch with 4 second release resulting in an 18% maximum stretch for 30 minutes at 37 °C. Cells were harvested in 200 µl lysis buffer/well RIPA buffer (Merck, UK, R0278) supplemented with 1% v/v phosphatase inhibitor-3 (Merck, UK, P0044) prior to stretch, immediately post and at 1,3,7 and 24 h post stretch. Protein concentration was determined by the Bio-Rad RCDC Protein Assay (BioRad, CA, USA, 5000121), and resulting lysates were stored for subsequent western blotting analysis.

#### Western blotting

Approximately 15–20 mg of wet weight muscle tissue was homogenised in 18 µL/mg RIPA buffer (Merck, UK, R0278) supplemented with 1% (v/v) phosphatase inhibitor-3 (Merck, UK, P0044). Samples were rotated for 90 min at 4 °C and centrifuged at 13,000 rpm for 15 min at 4 °C. The supernatant was collected, and protein concentration determined using the Bio-Rad RC DC Protein Assay (BioRad, UK, 5000121). The pellet was retained for 14-kDa actin fragment analysis. The 14kDa fragment is a cleavage product of actin and can be used as an indicator of myofibrillar protein breakdown. [16]. Lysates were subjected to SDS-PAGE using 10–12% gels on a mini-PROTEAN Tetra system (Bio-Rad, CA, USA). Proteins were transferred onto nitrocellulose membranes (ThermoFisher, UK, 88018) and blocked for 1 h with Tris-buffered saline with 5% (w/v) skimmed milk and 0.1% (v/v) Tween-20 (merck, UK, P1379). Membranes were incubated overnight with the primary antibodies against p-Akt (Ser^473^; 1:2,000; Cell Signalling Technologies, USA, 4060S) and p-p70S6K (Thr^389^; 1:500; Cell Signalling Technologies, USA, 2708S). For 14kDa fragment analysis, an AC-40 Actin clone antibody (1:500; Sigma Aldrich, UK, A4700) was used. This antibody also recognises the 42-kDa fragment, which served as a loading control with a shorter exposure time. For all other proteins, GAPDH was used as a loading control (1:1000, Cell Signalling Technologies, USA, 5174). Horseradish Peroxidase (HRP)-linked anti-mouse/rabbit secondary antibodies (Dako, Aglient, UK, P0260 and P0448 respectively) were used at 1:1500 for 2 h at room temperature. Blots were visualised using ECL Reagents (Geneflow, UK, K1-0098) and captured using a ChemiDoc MP imager (Bio-Rad).

#### Quantitative RT-PCR

Total RNA was isolated from 15 - 20 mg of muscle tissue using TRIzol® (ThermoFisher, UK, 15596026) and reversed transcribed to cDNA using Lunascript transcription system (New England BioLabs, MA, USA, E3010L). Primers and probes and internal controls were supplied as TaqMan gene expression assays (ThermoFisher, UK, 4331182) (Table 3). 18S rRNA served as the housekeeping gene and expression stability was confirmed across all conditions. Reference gene selection was based on an initial screen using a Human Endogenous Control Plate (ThermoFisher, UK, 4426696), followed by a validation of shortlisted candidates (18S rRNA, β-2-microglobulin (B2M), β-actin (ACTB), cancer susceptibility candidate 30 (CASC30), Processing of precursor 4 ribonuclease P/MRP subunit (POP4)) in a larger sample set by PCR. 18s rRNA showed the lowest coefficient of variation (2.18%) and highest expression, and was therefore selected as the reference gene. All reactions were performed in a 20 µL- volume containing 1 µL cDNA, 10 µL 2X Taqman Fast Mastermix (ThermoFisher, UK, 4444557), 8 µL nuclease-free water, and 1 µL primer/probe assay on an Applied Biosystem’s QuantStudio 3 instrument with the following conditions: 95 °C 15 s, followed by 40 cycles at 95 °C for 15 s and at 60 °C for 1 min. Target gene Ct values were normalised to the 18S rRNA house-keeping gene, and relative expression levels were calculated using the 2^-ΔΔCt^ method.

**Table 3:** Details of Taqman probes used within PCR experiments

Gene name	Full name	Taqman Probe Assay ID
MAFbx	Muscle atrophy F-box protein	Hs00369714_m1
MuRF-1	Muscle RING finger-1	Hs00822397_m1
MSTN	Myostatin	Hs00976237_m1
ActRIIB	Activin Receptor IIB	Hs00155658_m1
MYOG	Myogenin	Hs01072232_m1
MyoD	Myogenic differentiation 1	Hs02330075_g1
Myf5	Myogenic Factor 5	Hs00929416_m1
Pax7	Paired Box 7	Hs00242962_m1
18S rRNA	18-Svedberg ribosomal RNA	Hs99999901_m1

#### Availability of data

The datasets supporting the conclusions of this article are available in the University of Leicester Figshare repository (Watson, Emma (2025). ExTra CKD Biopsy Analysis. University of Leicester. Dataset. https://figshare.le.ac.uk/articles/dataset/_/30665759

#### Statistics

Data distribution was assessed using the Shapiro-Wilks test. Variables that were not normally distributed were log-transformed prior to analysis to meet the assumptions of parametric testing. All statistical analyses were performed on the transformed data; values are presented as back-transformed estimates to aid interpretation. All outcome variables were analysed using repeated-measures ANOVA. For in vivo exercise experiments, group (AE vs CE) was included as a between-subject factor and time (baseline, untrained, trained) as a within-subject factor. For in vitro stretch experiments, condition (CKD vs healthy control) was included as a between-subject factor and time (baseline, immediately post-stretch, 1 h, 3 h, 7 h, and 24 h) as a within-subject factor.

Where significant main effects or interactions were observed, Bonferroni-adjusted post-hoc tests were used to compare specific time points and conditions. All data are expressed as mean ± standard deviation. Statistical significance was accepted at *p* < 0.05. All statistical analysis was carried out using IBM SPSS Statistics (IBM, Chicago, IL) Version 24.

## Results

### Participant characteristics

Participant characteristics for the ExTra CKD and Explore CKD studies are presented in Tables 1 and 2, respectively. Baseline characteristics were well-matched between the exercise groups (AE vs. CE) in the ExTRA CKD trial (Table 1) and between the donor groups (CKD vs. HC) in the Explore CKD study (Table 2).

### The anabolic response to exercise in CKD

**Fig. 2:**
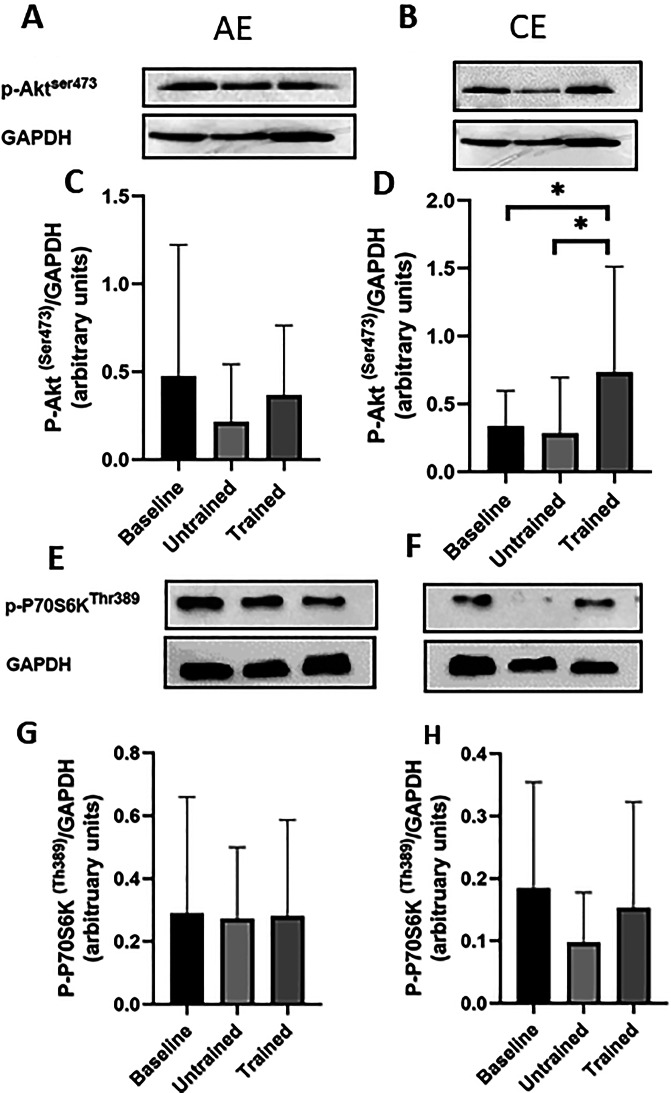
Changes in phosphorylation of Akt and P70S6K in skeletal muscle biopsies in response to unaccustomed and accustomed bouts of AE or CE. People with CKD undertook 12 weeks of either AE or CE training. Vastus lateralis muscle biopsies were collected at baseline, 24 h after a bout of unaccustomed exercise (untrained) and 24 h after a bout of accustomed exercise (Trained) in both groups. **A**) shows a representative western blot image for P-AktSer^473^ at baseline, untrained and trained time points with GAPDH that was used as a loading control in those people randomised to AE, and **B**) in those randomised CE. **C**-**D**) Histograms displaying densitometric data. **E**) shows a representative western blot image for *p*-70S6KThr^389^ at baseline, untrained and trained time points with GAPDH that was used as a loading control in those people randomised to AE and **F**) in those randomised to CE. For both proteins, AE *n* = 9, CE *n* = 8. Data are presented as mean±SD. * denotes *p* < 0.05. Abbreviations: AE, aerobic exercise, CE, combined exercise

Changes in phosphorylation of Akt and p70S6K are shown in Fig. 2. Akt phosphorylation is well documented to increase in the hours following exercise [17, 18], and is a key contributor to the anabolic response to exercise [19]. Repeated-measures two-way ANOVA revealed a significant main effect of time on Akt phosphorylation (Greenhouse-Geisser corrected *p* = 0.042), with no significant main effect of group (*p* = 0.53) and no significant group × time interaction (*p* = 0.098; Fig. 2A–D). Despite the overall effect of time, Bonferroni-adjusted post-hoc analyses demonstrated that Akt phosphorylation was not significantly altered from baseline at any individual time point following AE (*p* > 0.05). Similarly, a single bout of CE in the untrained state did not significantly change Akt phosphorylation from baseline (post-hoc *p* = 0.48). In contrast, following 12 weeks of CE training, post-hoc analysis revealed a significant 118% increase in Akt phosphorylation from baseline (post-hoc *p* = 0.02). This response was also significantly greater than that observed in the untrained state at the corresponding time point (post-hoc *p* = 0.02).

In contrast, p70S6K phosphorylation was not significantly changed by an acute bout of AE or CE in either the untrained or trained state (Fig. 2E–H). Repeated-measures two-way ANOVA revealed no significant main effect of time (Greenhouse-Geisser corrected *p* = 0.58), no significant main effect of group (*p* = 0.16), and no significant group × time interaction (*p* = 0.76) for p70S6K phosphorylation (Fig. 2E–H).

### The catabolic response to exercise in CKD

**Fig. 3:**
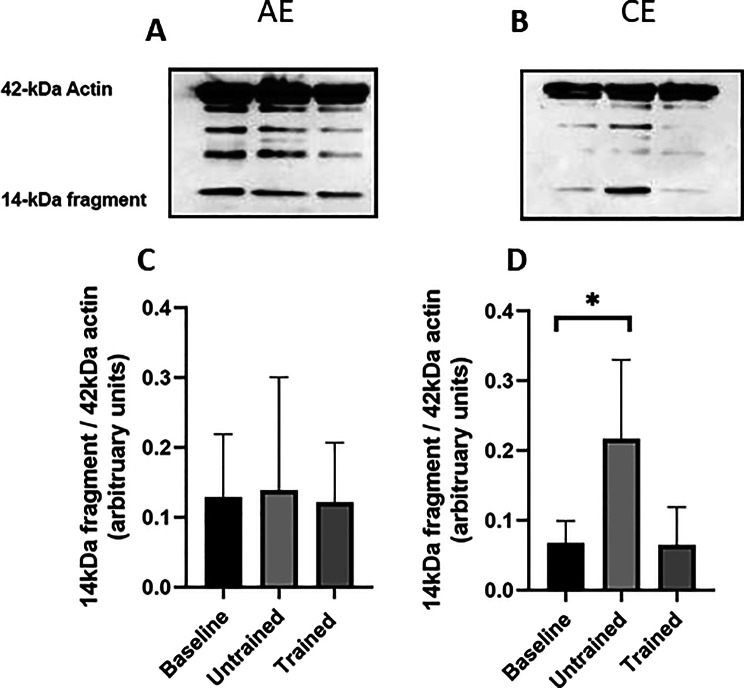
Abundance of the 14kDa actin fragment in skeletal muscle biopsies in response to unaccustomed and accustomed bouts of AE or CE. People with CKD undertook 12 weeks of either AE or CE training. Vastus lateralis muscle biopsies were collected at baseline, 24 h after a bout of unaccustomed exercise (untrained) and 24 h after a bout of accustomed exercise (Trained) in both groups. **A**) shows a representative full western blot image that are labelled to show the 42kDa and the 14kDa actin fragments from people within the AE group and **B**) CE group. Histograms in **C**-**D** show densitometric data. AE *n* = 6, CE *n* = 5. Data are presented as mean±SD. * denotes *p* < 0.05. Abbreviations: AE, aerobic exercise, CE, combined exercise

Changes in the 14-kDa actin fragment, a biomarker of skeletal muscle catabolism [16], are shown in Fig. 3. Repeated-measures two-way ANOVA revealed a significant main effect of time on 14-kDa fragment abundance (Greenhouse–Geisser corrected *p* = 0.040), with no significant main effect of group (*p* = 0.5) and no significant group × time interaction (Greenhouse–Geisser corrected *p* = 0.079).

Bonferroni-adjusted post-hoc analysis demonstrated that the abundance of the 14-kDa fragment remained unchanged at all time points following AE (*p* > 0.05). In contrast, an acute bout of CE in the untrained state resulted in a significant 253% increase in the 14-kDa fragment relative to baseline (post-hoc *p* = 0.04). This response was abrogated after 12 weeks of CE training, with fragment levels no longer differing from baseline (post-hoc *p* = 0.68).

**Fig. 4:**
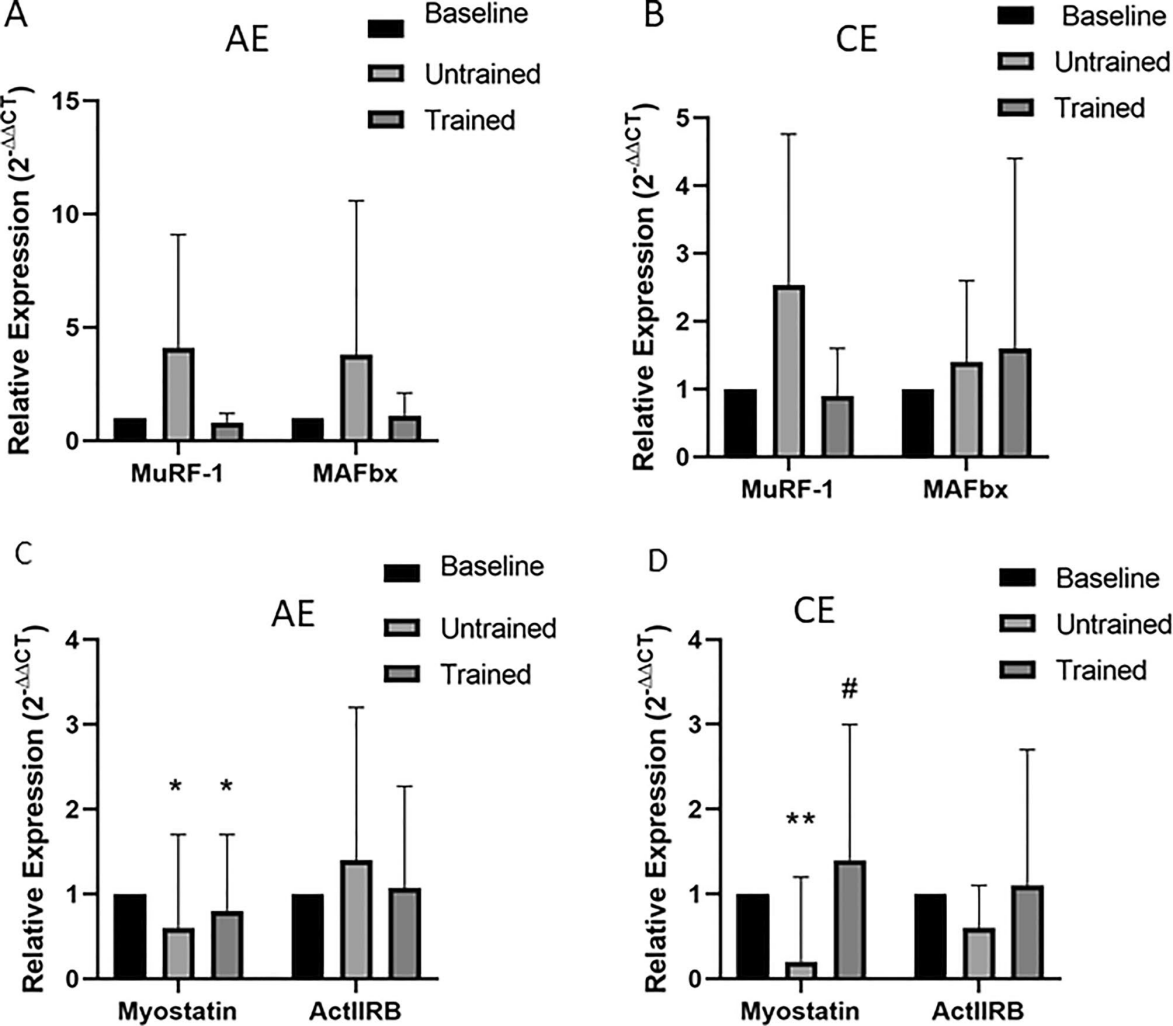
Changes in mRNA expression of genes related to atrophy processes in response to unaccustomed and accustomed bouts of AE or CE. People with CKD undertook 12 weeks of either AE or CE training. Vastus lateralis muscle biopsies were collected at baseline, 24 h after a bout of unaccustomed exercise (untrained) and 24 h after a bout of accustomed exercise (Trained) in both groups. mRNA expression of MuRF-1 and MAFbx were analysed by RT-PCR in the **A**) AE group, and **B**) CE group. Myostatin and ActIIRB were analysed by RT-PCR in the **C**) AE group, and **D**) CE group. Expression is displayed as relative change from baseline according to 2−ΔΔCt method and normalized to 18S rRNA. Data are presented as mean±SD. MuRF-1 AE *n* = 9, CE *n* = 8; MAFbx AE *n* = 8 CE = 10; Myostatin AE = 10, CE = 9; AC2BR AE = 10, CE = 9. * denotes *p* < 0.05 vs baseline, ** denotes *p* < 0.01 vs baseline, # denotes *p* < 0.05 vs untrained. Abbreviations: AE, aerobic exercise; CE, combined exercise

MuRF-1 and MAFbx are two muscle-specific E3 ligases, commonly used as markers of ubiquitin-proteasome system activity [20]. Repeated-measures two-way ANOVA revealed no significant main effect of time (MAFbx: Greenhouse–Geisser corrected *p* = 0.64; MuRF-1: *p* = 0.13), no significant main effect of group (MAFbx: *p* = 0.53; MuRF-1: *p* = 0. 61), and no significant group × time interaction (MAFbx: *p* = 0.704; MuRF-1: *p* = 0.461) following acute AE or CE (Fig. 4). Consistent with these findings, neither MuRF-1 nor MAFbx mRNA expression was significantly altered at any time point following exercise.

In contrast, the mRNA expression of myostatin, a potent negative regulator of muscle mass [21], was significantly altered over time. Repeated-measures two-way ANOVA revealed a significant main effect of time on myostatin mRNA expression (Greenhouse–Geisser corrected *p* < 0.001), with no significant main effect of group (*p* = 0.64) and no significant group × time interaction (*p* = 0.82; Fig. 4). Repeated-measures two-way ANOVA demonstrated no significant effects of time, group, or group × time interaction on ActIIRB mRNA expression (*p* > 0.05; Fig. 4).

### The myogenic response to exercise in CKD

**Fig. 5:**
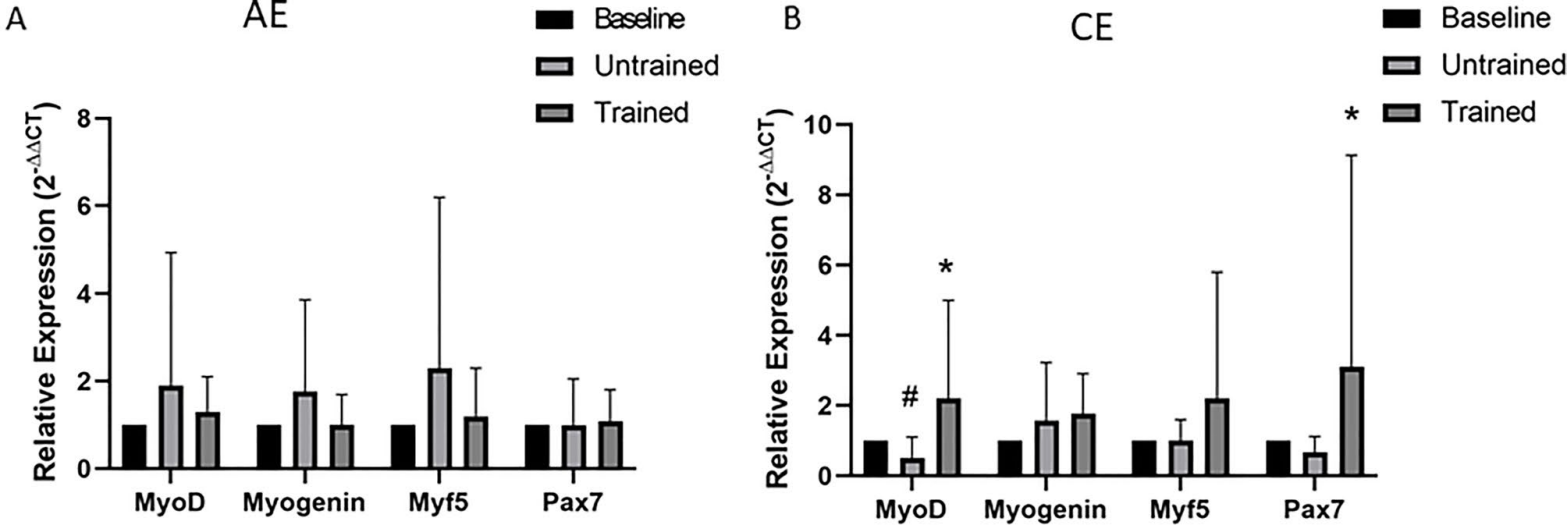
Changes in mRNA expression of genes related to myogenesis in response to unaccustomed and accustomed bouts of AE or CE. People with CKD undertook 12 weeks of either AE or CE training. Vastus lateralis muscle biopsies were collected at baseline, 24 h after a bout of unaccustomed exercise (untrained) and 24 h after a bout of accustomed exercise (Trained) in both groups. mRNA expression of genes within the myogenic pathways were analysed by RT-PCR in the **A**) AE group and **B**) CE group. Expression is displayed as relative change from baseline according to 2−ΔΔCt method and normalized to 18S rRNA. Data are presented as mean±SD. MyoD AE *n* = 10, CE *n* = 9; Myogenin AE *n* = 10, CE *n* = 9, Myf5 AE *n* = 8, CE *n* = 9; pax 7 AE *n* = 10, CE *n* = 8.* denotes *p* < 0.05 vs untrained. # denotes *p* = 0.05 vs baseline. Abbreviations: AE, aerobic exercise; CE, combined exercise

The mRNA expression of myogenic regulatory factors (Pax7, MyoD, Myogenin and Myf5), which coordinate muscle repair [22] are shown in Fig. 5. Specifically, Pax7 mRNA expression was assessed as an indicator of satellite cell related transcriptional activity and myogenic signalling. Following AE, repeated-measures two-way ANOVA revealed no significant main effects of time or group, and no group × time interactions for any of the measured transcripts (all *p* > 0.05).

In response to CE, no significant main effects of time, group, or group × time interactions were observed for Myogenin or Myf5 mRNA expression (all *p* > 0.05). In contrast, MyoD mRNA expression demonstrated a significant main effect of time (Greenhouse–Geisser corrected *p* = 0.016) and a significant group × time interaction (*p* = 0.031). Post-hoc analysis revealed a near-significant two-fold decrease in MyoD mRNA expression following CE in the untrained state (post-hoc = 0.05), which was reversed following training, resulting in a significant increase compared with the untrained condition (post-hoc *p* = 0.01). Pax7 mRNA expression showed no significant main effect of time or group × time interaction (*p* > 0.05).

### In vitro anabolic signalling response to mechanical stretch

**Fig. 6:**
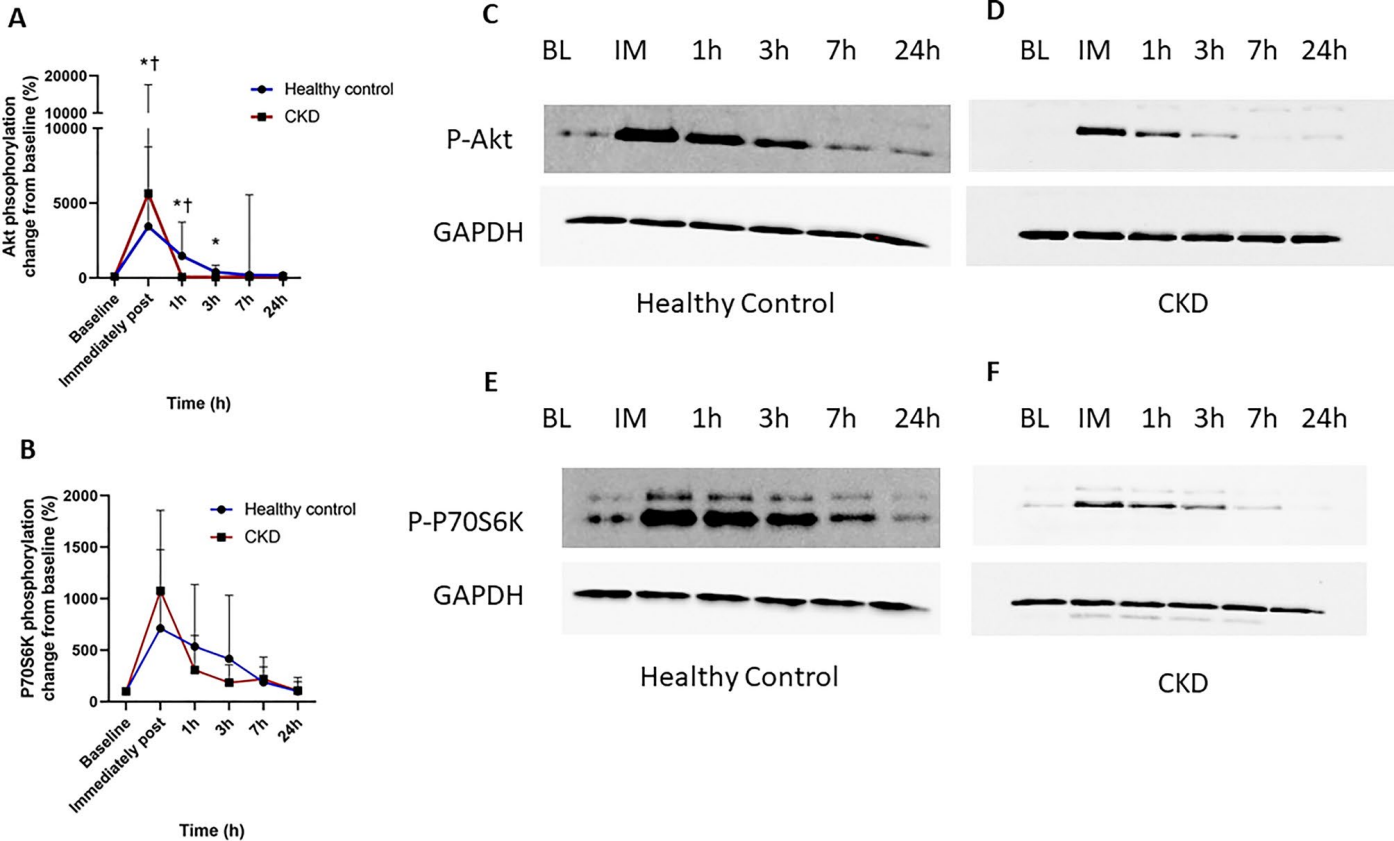
The effect of 18% cyclic stretch on the phosphorylation of Akt and P70S6K in primary skeletal muscle myotubes from healthy control or CKD donors. Myotubes were stretched on 6-well Flexcell culture plates for 30 minutes using a Flexcell F × 3000 system set to 2 second sine wave stretch with 4 second release. Cells were harvested for western blotting prior to stretch (baseline), immediately post, and at 1,3,7 and 24 h post stretch for the analysis of P-Akt and P-P70S6K. **A**) Time course of % change from baseline of P-Akt, *n* = 8 healthy control, *n* = 6 CKD, and **B**) P-P70S6K *n* = 7 healthy control, *n* = 5 CKD. Representative western blot images of **C**) P-Akt normalised to GAPDH in a healthy control donor and **D**) P-Akt normalised to GAPDH in a CKD donor **E**) P-P70S6K normalised to GAPDH in a healthy control donor and **F**) P-P70S6K normalised to GAPDH in a CKD donor. Data are presented as mean±SD. * Denotes *p* < 0.05 vs baseline in HC. † denotes *p* < 0.05 vs baseline in CKD. Abbreviations: BL, Baseline; CKD, chronic kidney disease; h, hour; IM, immediately post stretch

Passive mechanical stretch was used as an experimental model to isolate mechanical loading–induced signalling pathways. This approach does not replicate the metabolic, neural, or contractile components of active muscle contraction but allows targeted investigation of mechanosensitive responses in skeletal muscle cells. To examine the temporal dynamics of anabolic signalling in response to mechanical stretch, primary myoblasts from CKD and HC donors were subjected to mechanical stretch in vitro. Mechanical stretch induced a marked increase in Akt phosphorylation relative to baseline in both groups immediately post-stretch (CKD: +5651%, post-hoc *p* = 0.012; HC: +3437%, post-hoc *p* = 0.028; Fig. 6A–D). This elevation persisted at 1- hour post-stretch (CKD: +61%, *p* = 0.012; HC: +1472%, post-hoc *p* = 0.028). At 3- hours post-stretch, Akt phosphorylation remained elevated in the HC group (+388%, *p* = 0.046) but had returned to baseline in the CKD group (post-hoc *p* = 0.31). By 7- and 24-hours post-stretch, phosphorylation levels had returned to baseline in both groups (Fig. 6A). Repeated-measures two-way ANOVA revealed no significant main effect of time (Greenhouse–Geisser corrected *p* = 0.068) and no significant group × time interaction (*p* = 0.317), indicating that while transient post-stretch increases were observed, the overall temporal response to mechanical stretch did not differ between CKD and healthy control groups.

A similar phosphorylation pattern was observed for p70S6K (Fig. 6D–F). Mechanical stretch induced a significant increase in p70S6K phosphorylation from baseline immediately post-stretch in both groups (CKD: +1076%, post-hoc *p* = 0.014; HC +712%, post-hoc *p* = 0.038). In CKD cells, this elevation persisted at 1- hour (+306%, post-hoc *p* = 0.047) and 7- hours post-stretch (+219%, post-hoc *p* = 0.004), with a strong trend toward an increase at 3 hours (+185%, post-hoc *p* = 0.052). In contrast, no further time points showed significant increases from baseline in the HC group. Repeated-measures two-way ANOVA revealed a significant main effect of time on p70S6K phosphorylation (Greenhouse–Geisser corrected *p* = 0.002). The group × time interaction did not reach statistical significance (Greenhouse–Geisser corrected *p* = 0.052), indicating that while temporal differences were observed within groups, the overall response to mechanical stretch was broadly similar between CKD and healthy control cells.

## Discussion

This study offers new insights into the molecular responses to aerobic versus combined exercise in individuals with CKD, while also supporting our previous findings in a different cohort and exercise modality [10]. Here we showed the combined aerobic and resistance exercise, but not aerobic exercise alone, elicits beneficial molecular adaptations in skeletal muscle of individuals with CKD, in line with our hypothesis. Following 12-weeks of CE participants exhibited a significant restoration of anabolic signalling in response to exercise through increased Akt phosphorylation, activation of myogenic markers and attenuation of acute catabolic responses. Complementary in vitro stretch experiments demonstrated that skeletal muscle cells from people with CKD are capable of mounting anabolic responses, although these were more transient than those observed in healthy controls. While Akt and p70S6K phosphorylation typically peak within hours of exercise, the present study was designed to examine whether anabolic signalling persists at a later time point. In populations with anabolic resistance, sustained signalling beyond the immediate post-exercise window may be critical for cumulative muscle adaptation. Thus, absence or presence of signalling at 24 hours provides insight into the durability of the anabolic response rather than its peak magnitude.

Evidence suggests that individuals with CKD do not always exhibit the expected physiological or molecular adaptations to exercise, potentially reflecting impairments in the molecular signalling pathways that regulate skeletal muscle remodelling. For instance, several studies have reported that aerobic training does not consistently lead to increases in VO₂_peak_ in patients with CKD [23–25], a finding linked to a blunted activation of mitochondrial biogenesis pathways [8]. Additionally, it was previously reported that after a single bout of resistance exercise, the expected activation of the insulin signalling pathway was largely absent, but this response was restored after a training period [10] – a finding we have replicated here. A clear understanding of the molecular responses that underpin these adaptations to exercise is crucial for designing adjunct therapies that maximise the benefits of exercise for patients with CKD.

Skeletal muscle homeostasis is maintained through a finely regulated balance between protein synthesis and protein degradation, a process that becomes dysregulated with ageing and is further exacerbated in CKD by factors like insulin resistance and chronic inflammation [11–13]. This manifests as anabolic resistance, characterised by impaired activation of key signalling pathways, including Akt-mTOR, in response to mechanical or nutritional stimuli. Consistent with this, our previous work demonstrated a blunted anabolic response to acute exercise in untrained individuals with CKD, which could be partially restored following a period of training [10]. The current study confirms and extends these findings by demonstrating that combined exercise training also restores the capacity of the skeletal muscle to activate Akt signalling in response to an acute bout, supporting the notion that exercise training can somewhat recalibrate anabolic sensitivity in CKD, which is also seen in the context of ageing [26].

A major limitation of human muscle biopsy studies is the limited temporal resolution. As our in vivo samples were collected 24 hours post-exercise, crucial early molecular responses may have been missed. To address this gap, we conducted in vitro mechanical stretch experiments using primary myoblasts from both healthy controls and individuals with CKD. These cells are known to retain key aspects of their in vivo phenotype [15] and were matched for age, gender and physical activity levels (all had low physical activity levels), which allowed for more detailed interrogation of the temporal dynamics of mechanically induced anabolic signalling. Mechanical stretch robustly increased of Akt and p70S6K phosphorylation in cells from both groups, with peak activation occurring between 0 and 7 hours post-stretch and returning to baseline by 24 hours. This 24-hour result replicates our in vivo observation of no sustained change at that time point. Interestingly, there was no significant difference in Akt phosphorylation response between CKD and control cells across this timecourse. While a trend was observed for a more acute transient p70S6K phosphorylation in CKD cells compared to a more sustained response in controls. Although this did not reach statistical significance, possibly due to high inter-donor variability, it raises the possibility that anabolic resistance in CKD may reflect impaired maintenance rather than initiation of anabolic signalling. As each replicate represented a different donor, future studies using multiple replicates from the same donor could clarify these responses. Integrating the in vivo and in vitro data suggests that individuals with CKD are capable of mounting an anabolic response to exercise; however, this response appears to be relatively transient. Conducting an in vitro ‘training’ model using repeated bouts of mechanical stretch could offer valuable insights into how chronic loading influences the durability and magnitude of the anabolic signalling response in CKD patients.

An essential component of muscle adaptation is the activation of satellite cells for repair and regeneration [27]. In our study, AE did not stimulate changes in the expression of myogenic regulatory factors. In contrast, CE induced a more pronounced response after the training period, with significant upregulation of MyoD expression. Given that resistance exercise is a potent stimulus for muscle damage and repair, it is plausible that regenerative processes are more strongly activated by CE than by AE alone.

This study has several limitations. The timing of muscle biopsy collection restricted to a single time point 24 hours post-exercise limits the conclusions that can be drawn to a narrow temporal window. As such, early molecular events that may have occurred in the immediate hours following exercise could have been missed. This limitation was a key rationale for conducting the complementary in vitro study, which allowed for a more detailed examination of the temporal profile of anabolic signalling. It must be highlighted that whilst a good in vitro model, mechanical stretch of human primary muscle cells does not fully recapitulate exercise in vivo. It does not capture the complex interactions between hormonal changes, neural input or immune responses that can all affect the molecular response within the muscle. 2D cultured primary cells can also lose aspects of their tissue-level organisation and environmental context over time. Factors such as extracellular matrix composition, cell–cell interactions, and 3D architecture, which influence signal transduction in vivo. To better capture the dynamics of molecular responses to exercise in future in vivo studies, multiple biopsy time points across the acute post-exercise period should be considered. The biopsy component of the ‘ExTra CKD trial’ was exploratory in nature and not formally powered. The sample size was determined pragmatically based upon participant consent to optional biopsy procedures within the parent randomised controlled trial. A further limitation is the absence of a new baseline biopsy obtained after the training intervention. Exercise training has been shown to alter basal expression of genes and proteins involved in muscle remodelling, which may influence interpretation of post-exercise responses. Consequently, acute signalling responses in the present study are interpreted relative to the pre-training baseline, and changes following training may reflect both altered basal expression and altered responsiveness to exercise. This limitation reflects the practical constraints of conducting repeated muscle biopsies in individuals with CKD and should be addressed in future work employing more intensive sampling designs. Furthermore, normalisation to a loading control in our western blotting analysis rather than total target protein, reflects tissue limitations inherent to human biopsy studies and should be considered when interpreting phosphorylation data. Previous work has demonstrated that the order of aerobic and resistance exercise can influence acute post-exercise signalling responses [28, 29]. Although aerobic exercise was typically performed before resistance exercise in the present study, the order was not strictly standardised and may have contributed to variability in acute signalling outcomes.

In conclusion this study provides novel insight into the molecular responses of skeletal muscle to different modes of exercise in individuals with CKD. Using both in vivo muscle biopsy data and complementary in vitro models, we demonstrate that combined exercise training, but not aerobic training alone, can restore aspects of anabolic signalling, namely Akt phosphorylation, in skeletal muscle, supporting the notion that resistance-based modalities are necessary to elicit beneficial molecular adaptations in this population and confirming our earlier work. While the in vitro stretch model helped clarify the temporal dynamics of early signalling events, it also highlighted the complexity of translating findings across experimental models. Our findings suggest that individuals with CKD are capable of mounting an anabolic and myogenic response to exercise, but that this response may be blunted or short-lived without repeated loading. The observed changes in catabolic and regenerative markers further support the importance of exercise modality in shaping muscle adaptation and further validating the role of exercise training in reversing anabolic resistance in CKD. Future work should focus on longitudinal models, both in vitro and in vivo, to better understand how repeated loading influences anabolic signalling, and how these molecular responses relate to clinically meaningful improvements in muscle mass and function in CKD. Our findings here support the inclusion of a resistance component within exercise programmes for CKD.

## Electronic supplementary material

Below is the link to the electronic supplementary material.

Supplementary Material 1

Supplementary Material 2

## Data Availability

The datasets supporting the conclusions of this article are available in the University of Leicester Figshare repository (Watson, Emma (2025). ExTra CKD Biopsy Analysis. University of Leicester. Dataset. https://figshare.le.ac.uk/articles/dataset/_/30665759

## Abbreviations

18S rRNA: 18-Svedberg ribosomal RNA
ACTB: β-actin
ActRIIB: Activin Receptor IIB
AE: Aerobic exercise
ANOVA: Analysis of variance
B2M: β-2-microglobulin
BIA: Bioelectrical Impedance Analysis
BMI: Body mass index
BSA: Bovine Serum Albumin
CASC30: Cancer susceptibility candidate 30
CE: Combined exercise
CKD: Chronic kidney disease
DNA: Deoxyribonucleic acid
FBS: Foetal Bovine Serum
GAPDH: Glyceraldehyde-3-phosphate dehydrogenase
GM: Growth Media
H: hour
HC: Healthy controls
HRP: Horse Radish Peroxidase
kDa: Kilodalton
MAFbx: Muscle atrophy F-box protein
MSTN: Myostatin
MuRF-1: Muscle RING finger-1
MAMC: Mid-Arm Muscle Circumference
MyoD: Myogenic differentiation 1
MYOG: Myogenin
PAX7: Paired Box 7
POP4: Processing of precursor 4 ribonuclease P/MRP subunit
RIPA: Radioimmunoprecipitation assay buffer
RNA: Ribonucleic acid
SDS-PAGE: Sodium Dodecyl Sulfate-polyacrylamide Gel Electrophoresis
SGA: Subjective Global Assessment

## References

[1] Global, regional, and national burden of chronic kidney disease, 1990-2017. A systematic analysis for the Global Burden of Disease Study 2017. Lancet. 2020;395(10225):709–33. 10.1016/S0140-6736(20)30045-3 PMC7049905 32061315

[2] Moorthi RN, Avin KG. Clinical relevance of sarcopenia in chronic kidney disease. Curr Opin Nephrol Hypertens. 2017;26(3):219–28. 28198733 10.1097/MNH.0000000000000318 PMC5860815

[3] Pereira RA, et al. Sarcopenia in chronic kidney disease on conservative therapy: prevalence and association with mortality. Nephrol Dial Transpl. 2015;30(10):1718–25. 10.1093/ndt/gfv133 25999376

[4] Wilkinson TJ, et al. Prevalence and correlates of physical activity across kidney disease stages: an observational multicentre study. Nephrol Dial Transpl. 2021;36(4):641–49. 10.1093/ndt/gfz235 31725147

[5] Schmid D, Ricci C, Leitzmann MF. Associations of objectively assessed physical activity and sedentary time with all-cause mortality in US adults: the NHANES study. PLoS One. 2015;10(3):e0119591. 10.1371/journal.pone.0119591 PMC4358950 25768112

[6] Baker LA, et al. Clinical practice guideline exercise and lifestyle in chronic kidney disease. BMC Nephrol. 2022;23(1):75. 35193515 10.1186/s12882-021-02618-1 PMC8862368

[7] Hu H, et al. Availability of exercise program, perceived exercise benefits and barriers, and exercise habits in maintenance hemodialysis patients: a multicenter cross-sectional study. J Ren Nutr. 2025;35(5):568–77. 40280376 10.1053/j.jrn.2025.03.009

[8] Watson EL, et al. Reductions in skeletal muscle mitochondrial mass are not restored following exercise training in patients with chronic kidney disease. Faseb J. 2020;34(1):1755–67. 31914685 10.1096/fj.201901936RR

[9] Watson EL, et al. Inflammation and physical dysfunction: responses to moderate intensity exercise in chronic kidney disease. Nephrol Dial Transpl. 2022;37(5):860–68. 10.1093/ndt/gfab333 35090033

[10] Watson EL, et al. The effect of resistance exercise on inflammatory and myogenic markers in patients with chronic kidney disease. Front Physiol. 2017;8:541. 28804461 10.3389/fphys.2017.00541 PMC5532513

[11] Wang XH, Mitch WE. Muscle wasting from kidney failure-a model for catabolic conditions. Int J Biochem Cell Biol. 2013;45(10):2230–38. 23872437 10.1016/j.biocel.2013.06.027 PMC3919551

[12] Wang XH, et al. Exercise ameliorates chronic kidney disease-induced defects in muscle protein metabolism and progenitor cell function. Kidney Int. 2009;76(7):751–59. 19641484 10.1038/ki.2009.260 PMC3835682

[13] Bailey JL. Insulin resistance and muscle metabolism in chronic kidney disease. ISRN Endocrinol. 2013;2013:329606. 23431467 10.1155/2013/329606 PMC3575670

[14] Watson EL, et al. Twelve-week combined resistance and aerobic training confers greater benefits than aerobic training alone in nondialysis CKD. Am J Physiol Renal Physiol. 2018;314(6):F1188–f1196. 10.1152/ajprenal.00012.2018 29412705

[15] Baker LA, et al. Establishment and characterisation of primary skeletal muscle cell cultures from patients with advanced chronic kidney disease. bioRxiv. 2020;2020.11.16.384263.

[16] Workeneh BT, et al. Development of a diagnostic method for detecting increased muscle protein degradation in patients with catabolic conditions. J Am Soc Nephrol. 2006;17(11):3233–39. 17005936 10.1681/ASN.2006020131

[17] Deshmukh A, et al. Exercise-induced phosphorylation of the novel Akt substrates AS160 and filamin a in human skeletal muscle. Diabetes. 2006;55(6):1776–82. 16731842 10.2337/db05-1419

[18] Sakamoto K, et al. Exercise regulates Akt and glycogen synthase kinase-3 activities in human skeletal muscle. Biochem Biophys Res Commun. 2004;319(2):419–25. 15178423 10.1016/j.bbrc.2004.05.020

[19] Schiaffino S, et al. Mechanisms regulating skeletal muscle growth and atrophy. Febs J. 2013;280(17):4294–314. 23517348 10.1111/febs.12253

[20] Bodine SC, Baehr LM. Skeletal muscle atrophy and the E3 ubiquitin ligases MuRF1 and MAFbx/atrogin-1. Am J Physiol Endocrinol Metab. 2014;307(6):E469–84. 10.1152/ajpendo.00204.2014 PMC4166716 25096180

[21] Elkina Y, et al. The role of myostatin in muscle wasting: an overview. J Cachexia Sarcopenia Muscle. 2011;2(3):143–51. 21966641 10.1007/s13539-011-0035-5 PMC3177043

[22] O’Sullivan TF, Smith AC, Watson EL. Satellite cell function, intramuscular inflammation and exercise in chronic kidney disease. Clin Kidney J. 2018;11(6):810–21. 30524716 10.1093/ckj/sfy052 PMC6275451

[23] Graham-Brown MPM, et al. A randomized controlled trial to investigate the effects of intra-dialytic cycling on left ventricular mass. Kidney Int. 2021;99(6):1478–86. 34023029 10.1016/j.kint.2021.02.027

[24] Watson EL, et al. Progressive resistance exercise training in CKD: a feasibility study. Am J Kidney Dis. 2015;66(2):249–57. 25533601 10.1053/j.ajkd.2014.10.019

[25] Greenwood SA, et al. Effect of exercise training on estimated GFR, vascular health, and cardiorespiratory fitness in patients with CKD: a pilot randomized controlled trial. Am J Kidney Dis. 2015;65(3):425–34. 25236582 10.1053/j.ajkd.2014.07.015

[26] Breen L, Phillips SM. Skeletal muscle protein metabolism in the elderly: interventions to counteract the ‘anabolic resistance’ of ageing. Nutr Metab (Lond). 2011;8:68. 21975196 10.1186/1743-7075-8-68 PMC3201893

[27] Snijders T, et al. Satellite cells in human skeletal muscle plasticity. Front. Physiol. 2015;6:2015. 10.3389/fphys.2015.00283 PMC4617172 26557092

[28] Coffey VG, et al. Effect of consecutive repeated sprint and resistance exercise bouts on acute adaptive responses in human skeletal muscle. Am J Physiol Regul Integr Comp Physiol. 2009;297(5):R1441–51. 10.1152/ajpregu.00351.2009 19692661

[29] Coffey VG, et al. Consecutive bouts of diverse contractile activity alter acute responses in human skeletal muscle. J Appl Physiol (1985). 2009;106(4):1187–97. 19164772 10.1152/japplphysiol.91221.2008

