# Supplementary material for: Anabolic and catabolic responses to different modes of exercise in patients with chronic kidney disease
Source: BMC Nephrol. 2026 Mar 13;27:252. doi: 10.1186/s12882-026-04891-4 (PMC13097932; doi:10.1186/s12882-026-04891-4)
Supplement: Supplementary file 1 — Supplementary Material 1 [file 12882_2026_4891_MOESM1_ESM.pptx]

## Slide 1
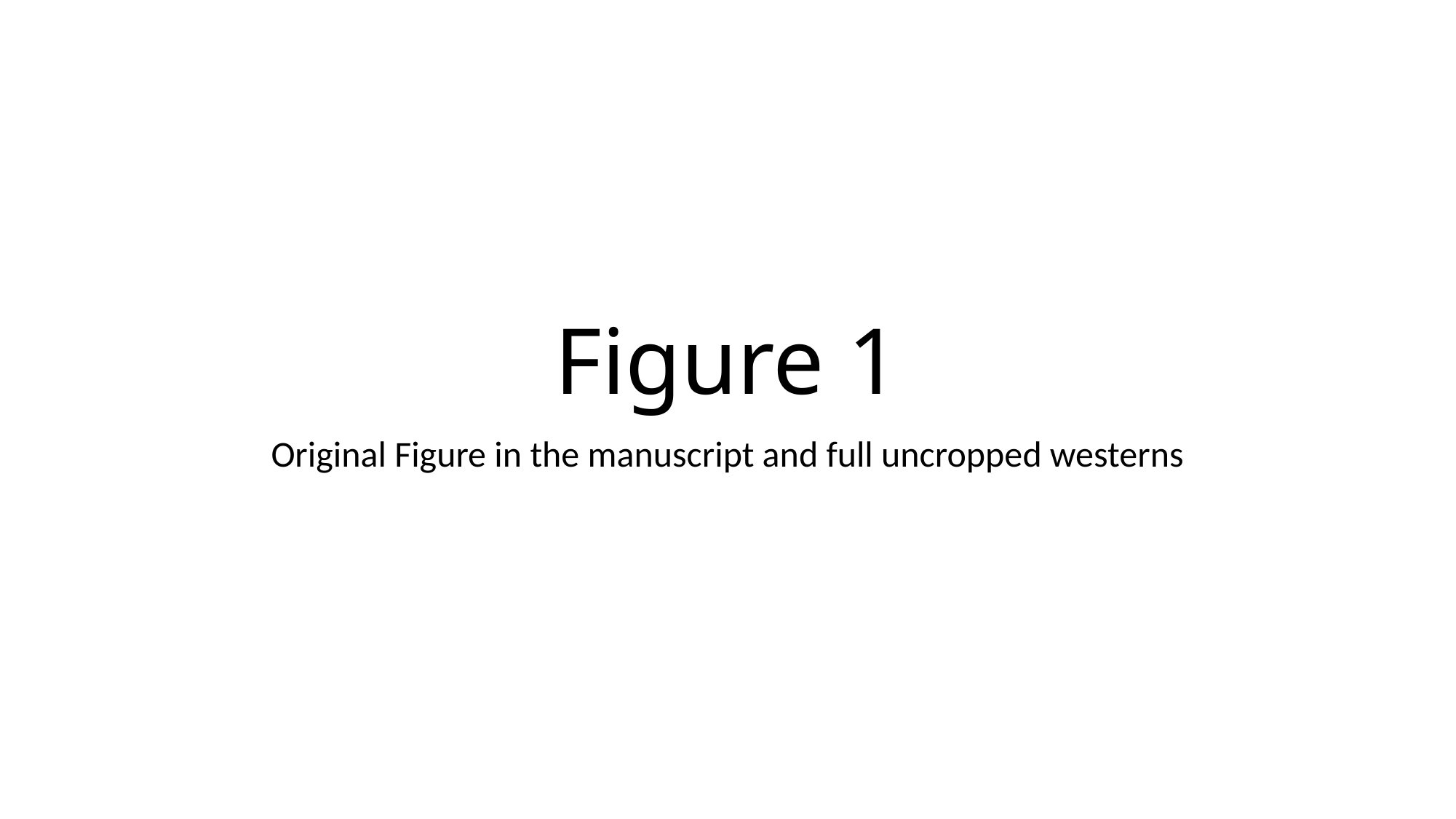

# Figure 1
Original Figure in the manuscript and full uncropped westerns

## Slide 2
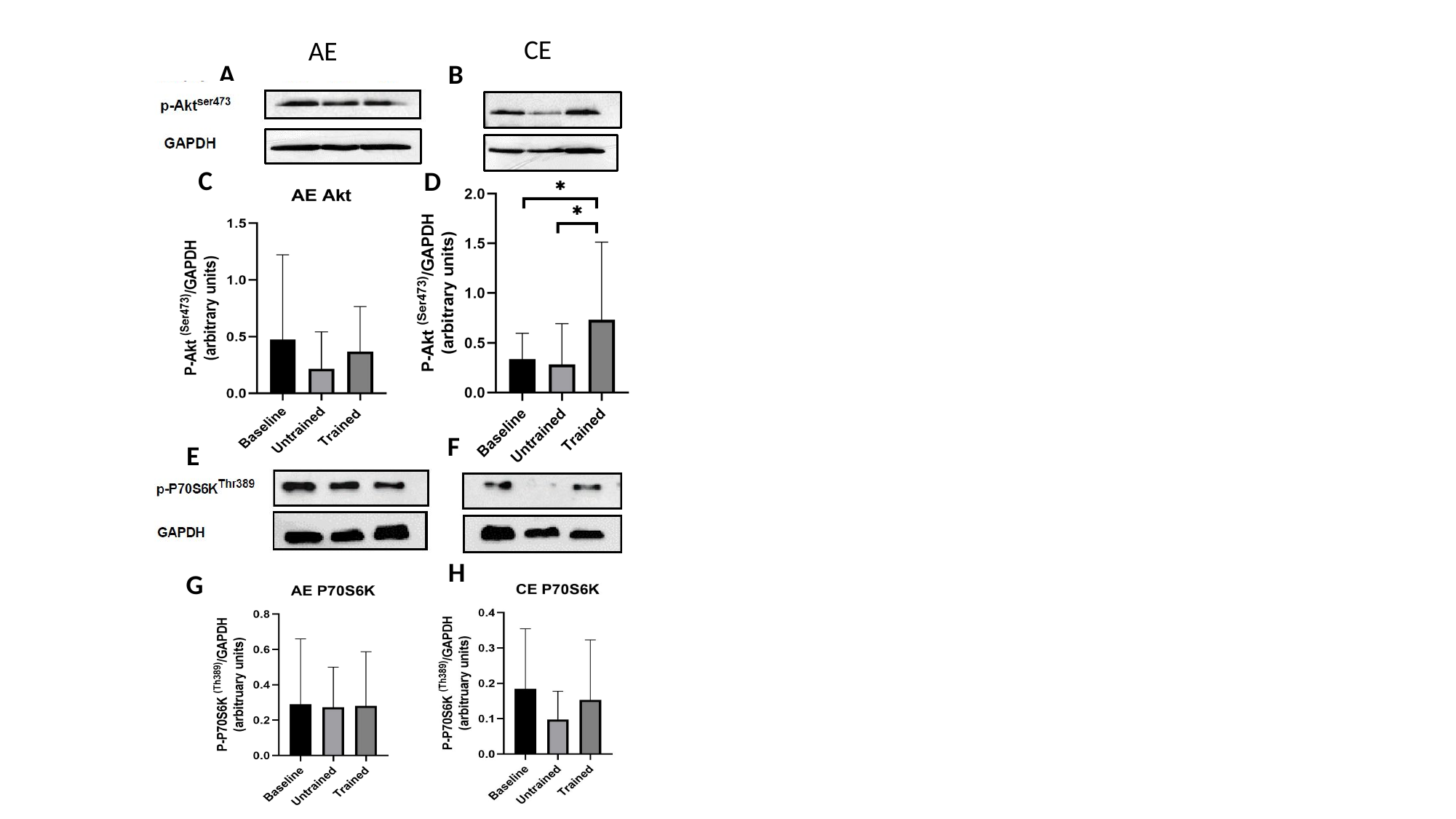

CE
AE
A
B
C
D
F
E
H
G

## Slide 3
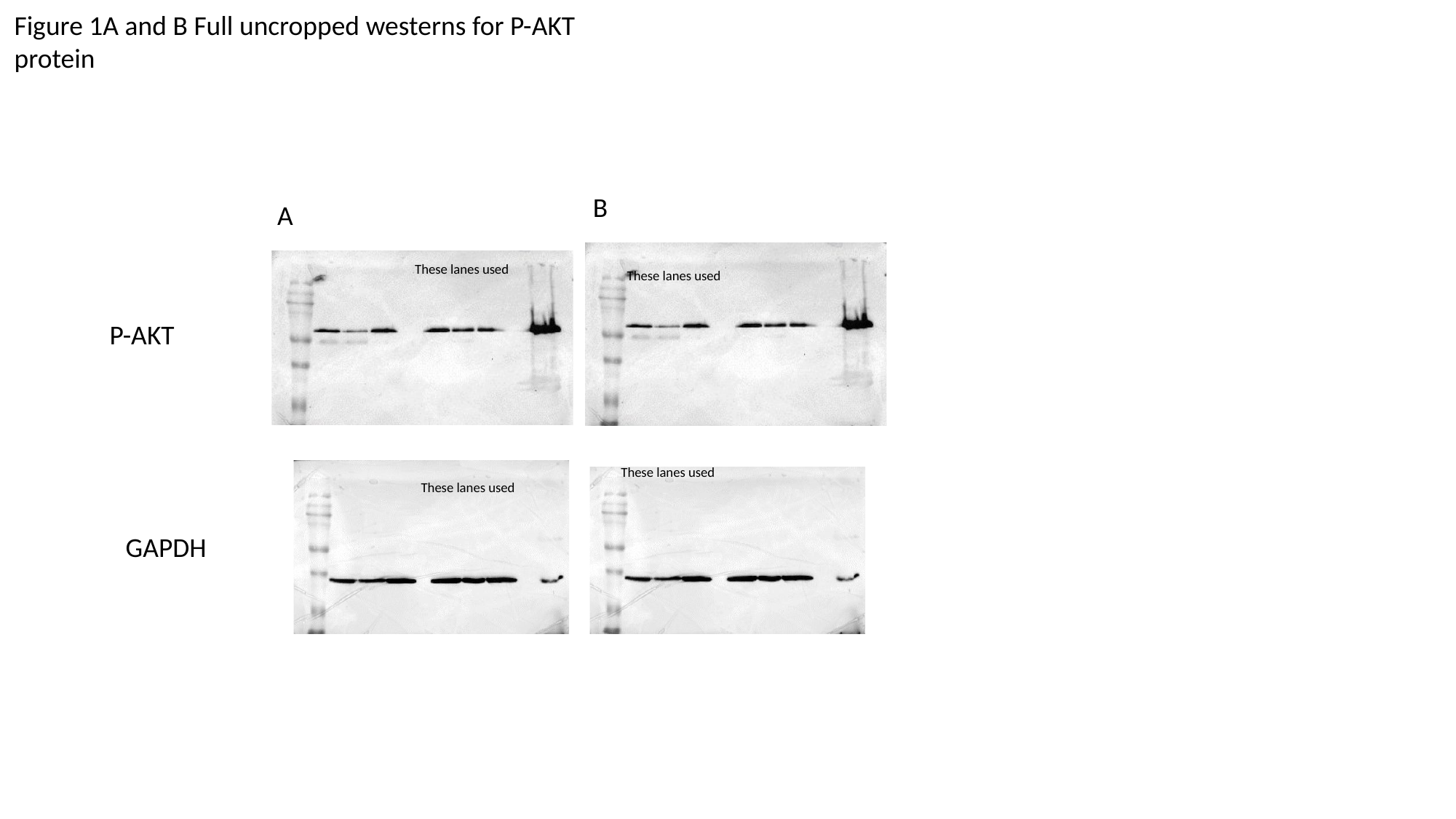

Figure 1A and B Full uncropped westerns for P-AKT protein
B
A
These lanes used
These lanes used
P-AKT
These lanes used
These lanes used
GAPDH

## Slide 4
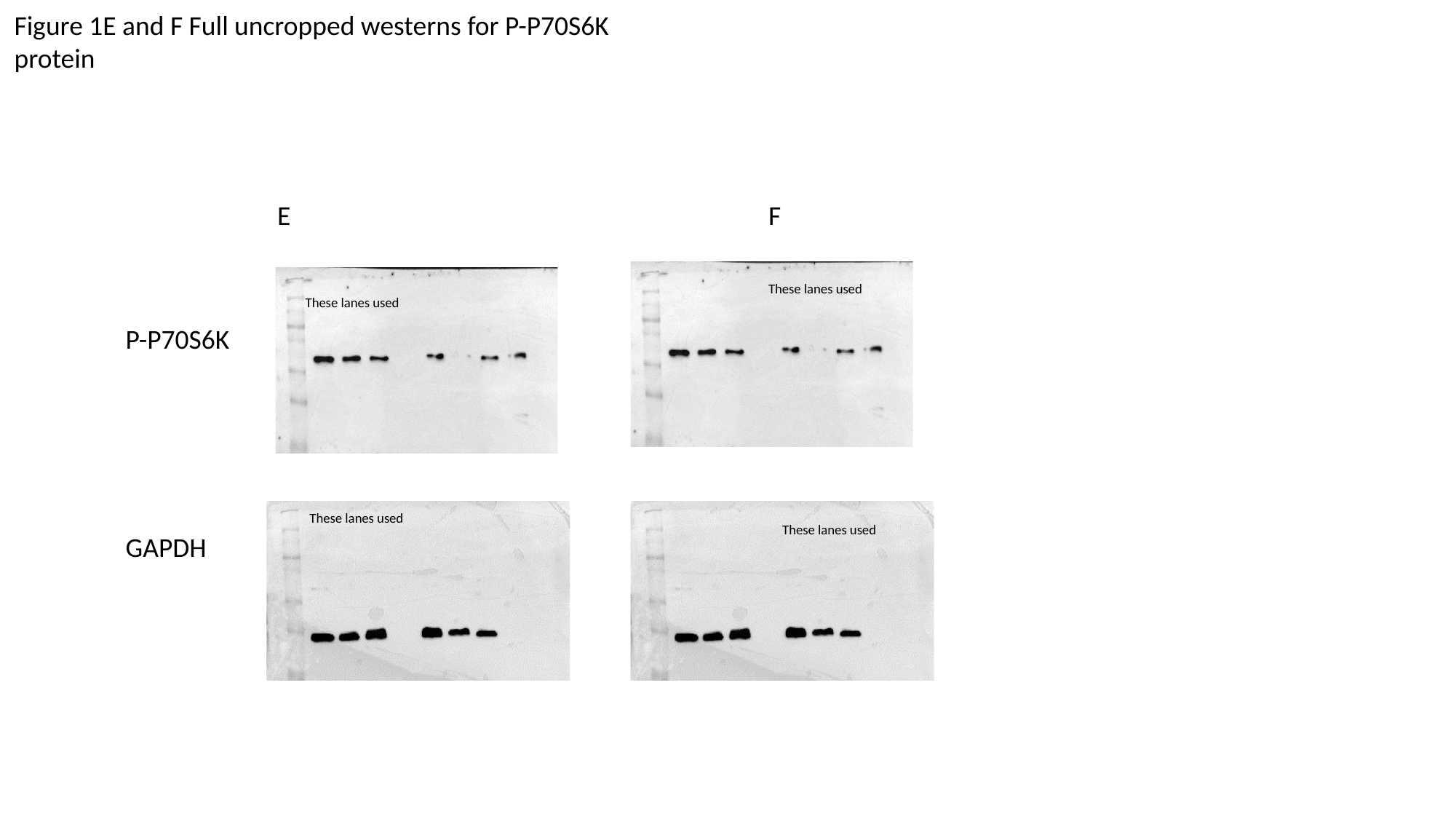

Figure 1E and F Full uncropped westerns for P-P70S6K protein
E
F
These lanes used
These lanes used
P-P70S6K
These lanes used
These lanes used
GAPDH

## Slide 5
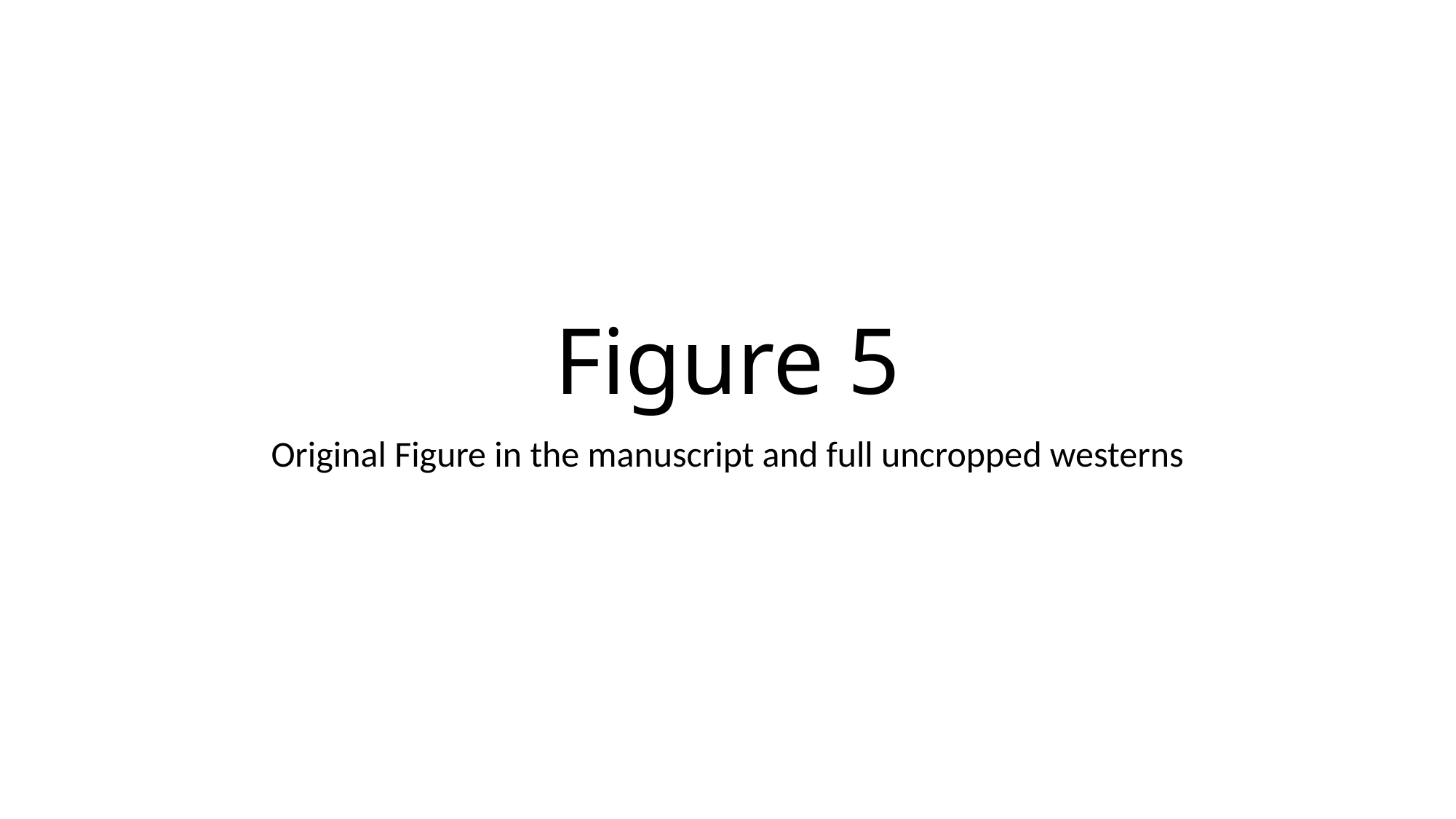

# Figure 5
Original Figure in the manuscript and full uncropped westerns

## Slide 6
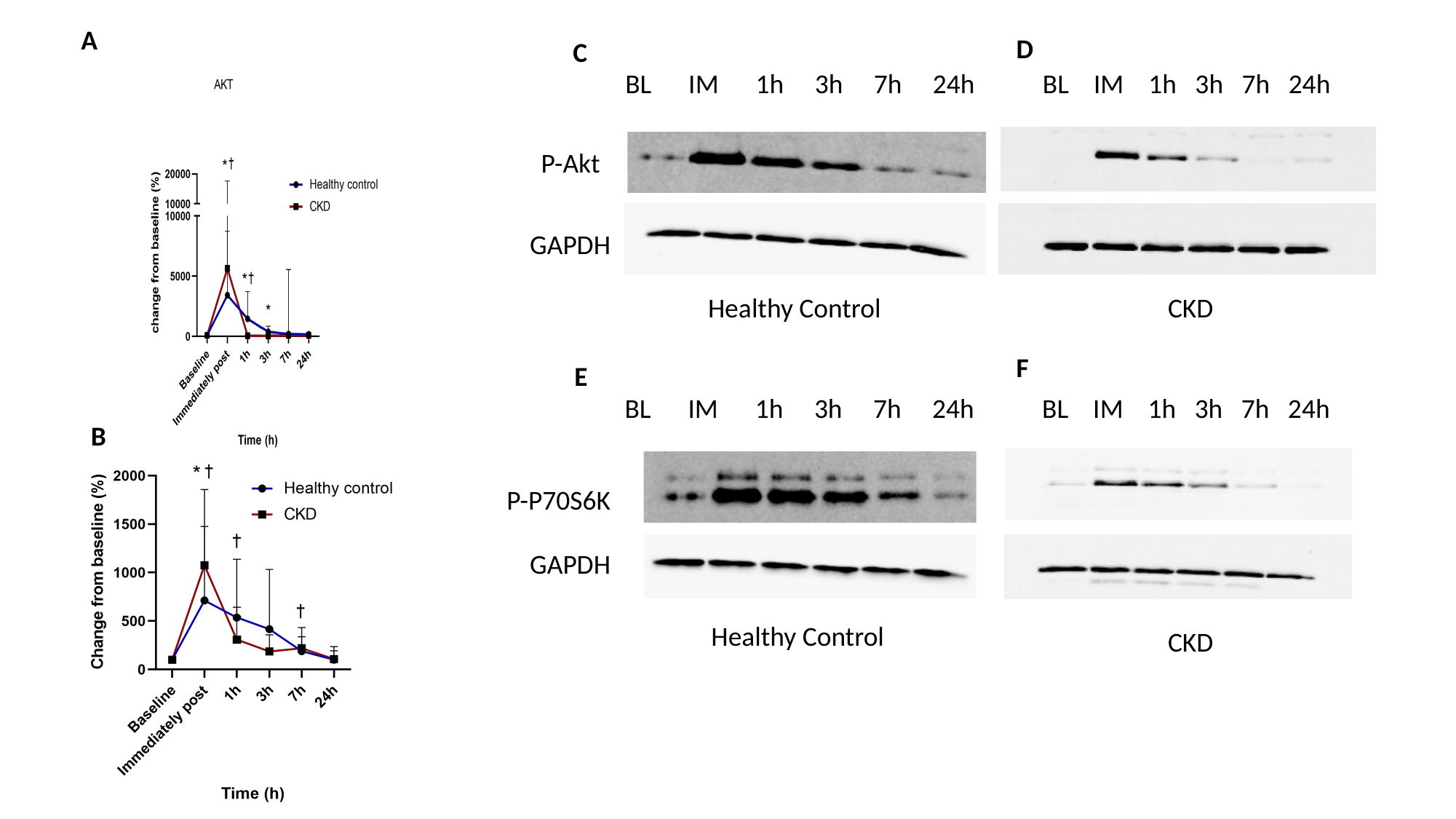

A
D
C
BL IM 1h 3h 7h 24h BL IM 1h 3h 7h 24h
P-Akt
GAPDH
Healthy Control
CKD
F
E
BL IM 1h 3h 7h 24h BL IM 1h 3h 7h 24h
B
P-P70S6K
GAPDH
Healthy Control
CKD

## Slide 7
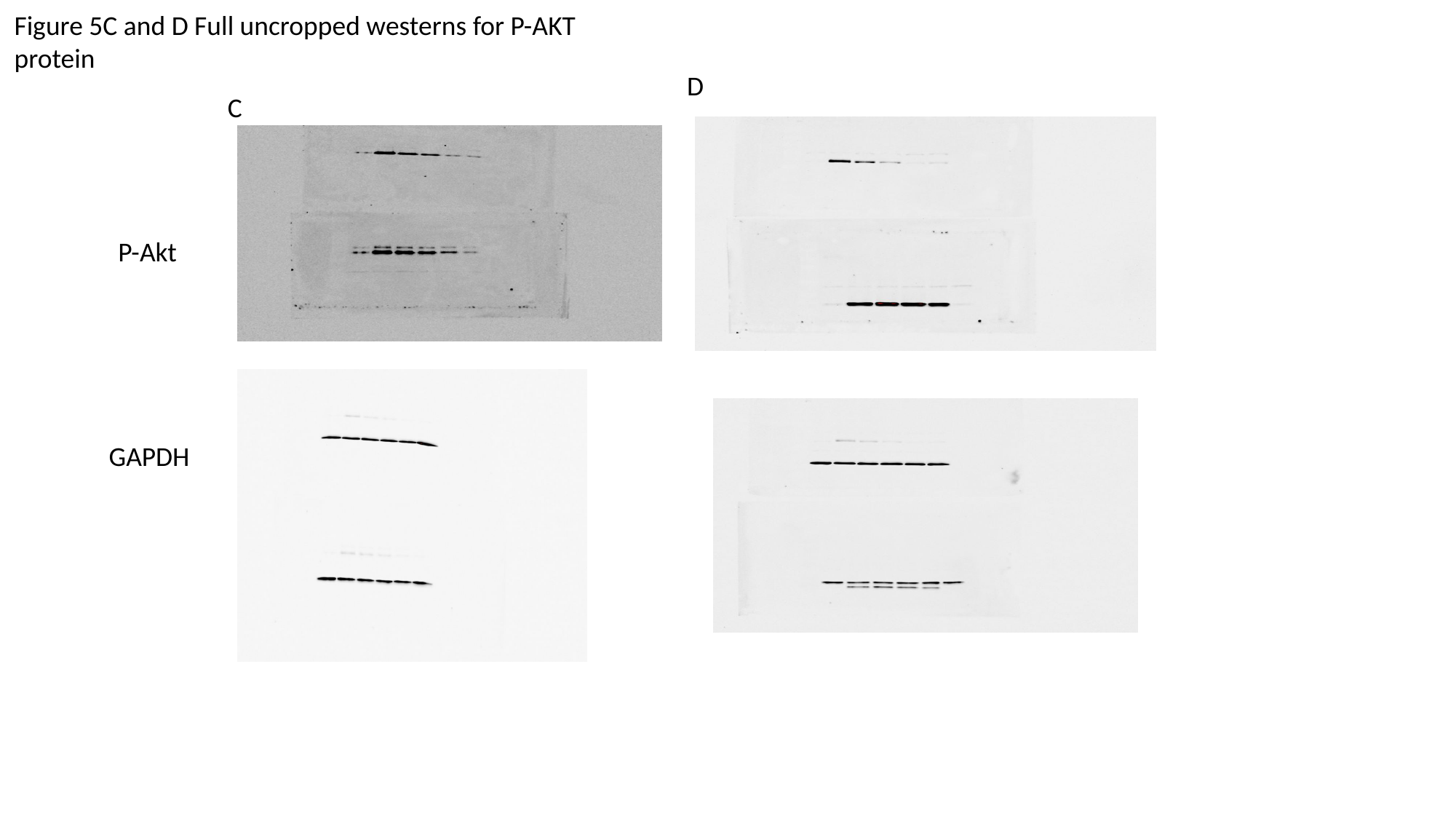

Figure 5C and D Full uncropped westerns for P-AKT protein
D
C
P-Akt
GAPDH

## Slide 8
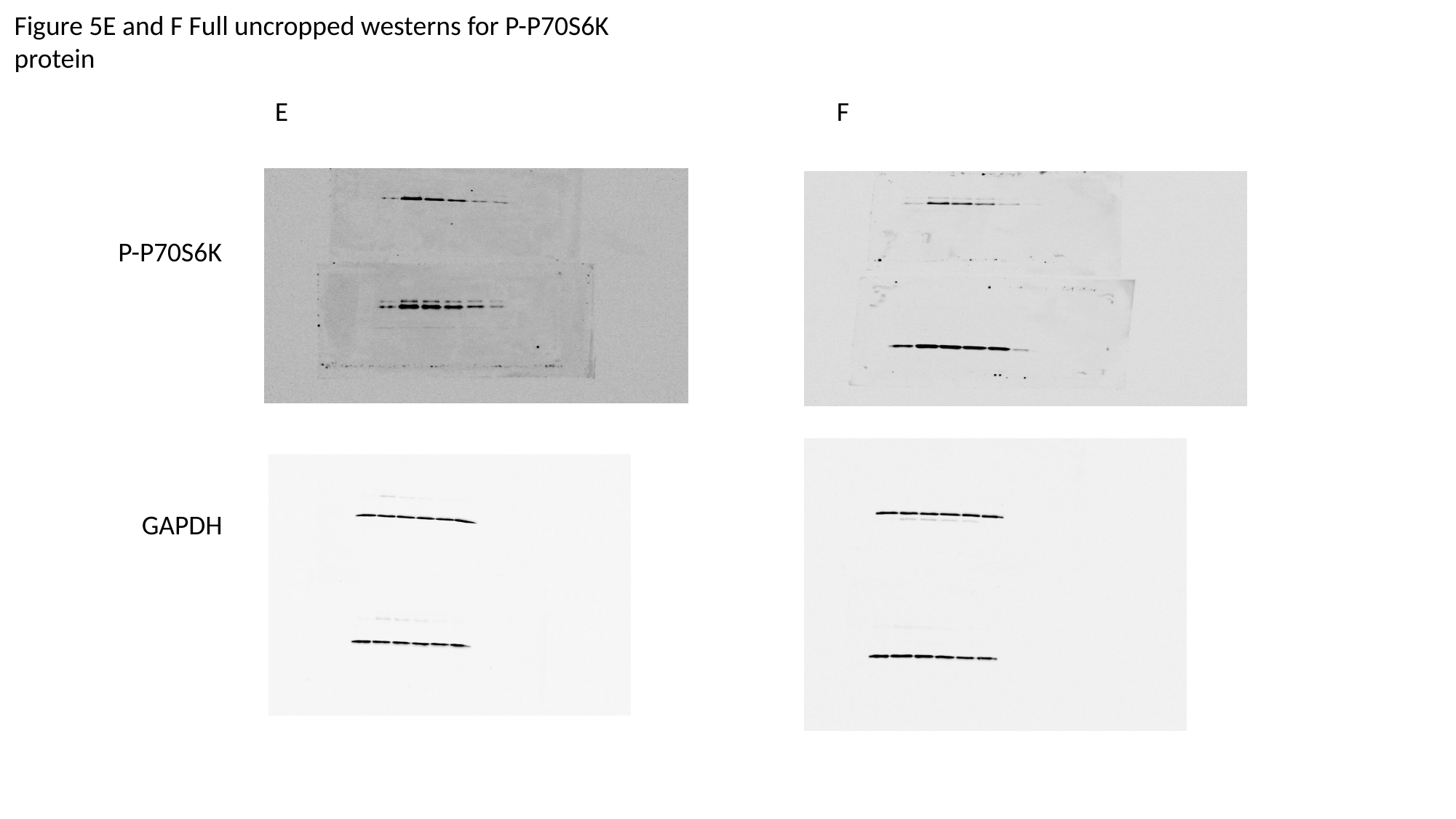

Figure 5E and F Full uncropped westerns for P-P70S6K protein
E
F
P-P70S6K
GAPDH
